# Transport coefficients in dense active Brownian particle systems: mode-coupling theory and simulation results

**DOI:** 10.1140/epje/s10189-021-00039-4

**Published:** 2021-03-11

**Authors:** Julian Reichert, Leon F. Granz, Thomas Voigtmann

**Affiliations:** 1 Institut für Materialphysik im Weltraum, Deutsches Zentrum für Luft- und Raumfahrt (DLR), 51170 Cologne, Germany; 2 Department of Physics, Heinrich-Heine Universität Düsseldorf, Universitätsstr. 1, 40225 Düsseldorf, Germany

## Abstract

**Abstract:**

We discuss recent advances in developing a mode-coupling theory of the glass transition (MCT) of two-dimensional systems of active Brownian particles (ABPs). The theory describes the structural relaxation close to the active glass in terms of transient dynamical density correlation functions. We summarize the equations of motion that have been derived for the collective density-fluctuation dynamics and those for the tagged-particle motion. The latter allow to study the dynamics of both passive and active tracers in both passive and active host systems. In the limit of small wave numbers, they give rise to equations of motion describing the mean-squared displacements (MSDs) of these tracers and hence the long-time diffusion coefficients as a transport coefficient quantifying long-range tracer motion. We specifically discuss the case of a single ABP tracer in a glass-forming passive host suspension, a case that has recently been studied in experiments on colloidal Janus particles. We employ event-driven Brownian dynamics (ED-BD) computer simulations to test the ABP-MCT and find good agreement between the two for the MSD, provided that known errors in MCT already for the passive system (i.e., an overestimation of the glassiness of the system) are accounted for by an empirical mapping of packing fractions and host-system self-propulsion forces. The ED-BD simulation results also compare well to experimental data, although a peculiar non-monotonic mapping of self-propulsion velocities is required. The ABP-MCT predicts a specific self-propulsion dependence of the Stokes–Einstein relation between the long-time diffusion coefficient and the host-system viscosity that matches well the results from simulation. An application of ABP-MCT within the integration-through transients framework to calculate the density-renormalized effective swim velocity of the interacting ABP agrees qualitatively with the ED-BD simulation data at densities close to the glass transition and quantitatively for the full density range only after the mapping of packing fractions employed for the passive system.

**Graphic abstract:**

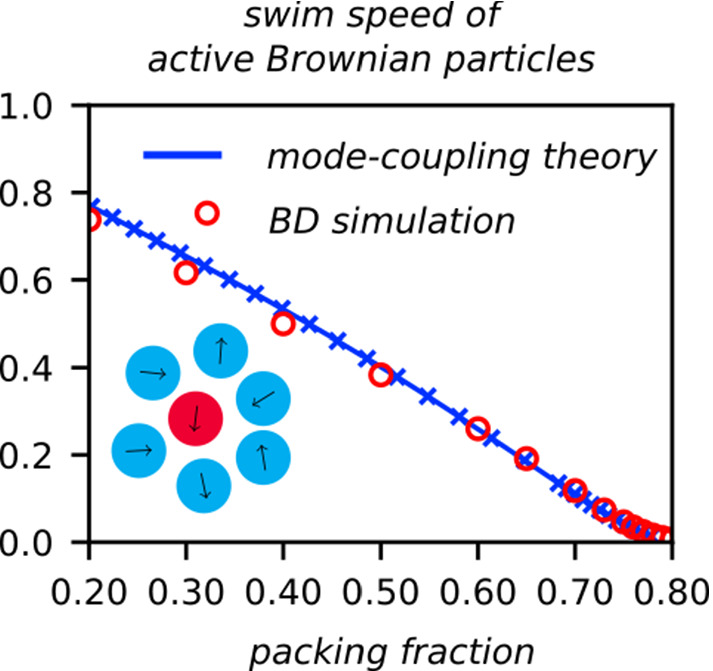

## Introduction

The study of transport phenomena far from equilibrium is a current exciting topic in statistical physics. One class of non-equilibrium systems is provided by living matter, defined as those biological systems where on the microscopic level, some mechanism is present to convert energy supplied by some fuel or food into directed motion. In microswimmer suspensions, these “active” or “self-propelled” entities are of some ${\upmu }\mathrm{m}$ in size and are thus subject to both thermal-equilibrium fluctuations that cause Brownian motion, and a motility that is caused by their non-equilibrium driving forces [1–3]. This alone causes an interesting interplay of dynamical effects; even more intriguing is this interplay in systems of interacting microswimmers, or in systems where microswimmers interact with ordinary “passive” Brownian particles.

Detailed experimental studies of interacting microswimmers are possible in colloidal suspensions of Janus particles [4–6]. These are generally particles that have two chemically different sides, such that a specifically designed interaction with the solvent can trigger phoretic forces causing motility. A specific example are colloidal particles coated with a light-absorbing surface in a suspension where local heating causes reversible microscale phase separation in the solvent [7, 8]. This model system has been studied extensively [6, 9]. It has also, together with computer simulations, established one of the most remarkable effects that appears in the moderately dense suspension of active particles, viz. that of motility-induced phase separation (MIPS) [6, 10]. It is generally believed that the equilibrium direct interaction between the particles is hard-sphere like, and hence, the observation of phase separation (or cluster formation) in a system with no apparent attractive interactions is rather striking.

More recently, experimental research has turned to very dense systems close to dynamical arrest at a glass transition and to the interaction of active particles with viscoelastic surroundings. We refer to a recent review by Janssen for an excellent overview [11]. Such situations might be closer to biophysical relevance, because most bio-relevant active particles tend to move in environments that are “crowded.” In particular, in experiment, the motion of a single active Janus particle in a suspension of passive particles has been studied through its mean-squared displacement (MSD) [12]. There emerges an interesting sequence of both sub-diffusive and superdiffusive motion, which signals a competition between dynamical arrest and persistent active motion.

Theoretical modeling of microswimmers proceeds via various model systems, among them that of active Brownian particle (ABP) [13–19]. In this model, Brownian translational and rotational diffusion is supplemented by a fixed self-propulsion velocity that causes the particles to move persistently with a fixed velocity in the direction of their (changing) orientation. Although in the dilute limit, many of the different models to implement active motion (such as the active Ornstein–Uhlenbeck particle (AOUP) model and related [20–28]) are roughly equivalent [29], they differ in the treatment of the coupling of orientational motion to self-propelled translational motion and in the treatment of Brownian to active forces. In particular, it is not evident whether the effective treatment of persistent motion that is encoded in these models is justified in very dense systems: it will be once the length scale of typical swimming motion before a particle loses memory of its initial orientation is small enough; yet one easily imagines that in a dense suspension, the small interparticle distance introduces a length scale that will interfere in subtle ways with the persistence length.

This rationale prompted us to develop a mode-coupling theory of the glass transition(MCT) to describe the approach to dynamical arrest in a dense ABP system, starting from the full orientation-resolved equations of motion. While arguably more complicated than other approaches, this mode-coupling theory for active Brownian particles (ABP-MCT) proved capable of describing states of dynamical arrest that depend on both the strength and the persistence of self-propelled motion [30]. More recently, we have extended this theory to include also equations of motion for the tagged-particle dynamics [31] and, based on that, the MSD of tracer particles [32]. This includes the case of tracers of different activity than that of the host system, and allows a more direct comparison of the theory to experiment, including the prediction of the interplay of sub- and superdiffusive motion that is not evidently present in orientation-averaged descriptions.

In the present contribution, we summarize these recent developments of ABP-MCT, and we provide a direct comparison of the theory to event-driven Brownian dynamics (ED-BD) computer simulations of ABP systems. Establishing the link between theory and simulation, we further extend to compare also the simulations to the experimental data of Ref. [12], to establish the extent to which this experimental model system can be taken as a realization of hard-sphere-like ABP.

A further specific point of ABP-MCT is that it is based on the integration-through transients (ITT) approach to the calculation of non-equilibrium transport coefficients in driven systems. Within ITT, one derives generalized Green–Kubo relations that link these transport coefficients to specific, microscopically defined, transient dynamical correlation functions. Here, the term transient correlation function is taken to mean those dynamical correlation functions that are obtained from averages over the equilibrium ensemble, but where the observables are propagated using the full non-equilibrium dynamics of the system. MCT-like approximations to these correlation functions provide first-principle predictions of the non-equilibrium transport coefficients.

In the case of ABP, the perhaps most interesting application in the dense system is that of the effective swim velocity: even though each ABP is supplied with a fixed self-propulsion velocity $v_0$, on a coarse-grained level the average motion of the particles is slowed down due to interactions, to a density-dependent velocity $v(\phi )\le v_0$. This quantity is a fundamental quantity for theories of MIPS [33–36], and in fact ITT is one of the few systematic approaches to calculate it from the microscopic equations of motion.

The paper is structured as follows: in Sect. 2.1, we first outline the ABP-MCT, followed by a derivation of the ITT expression for the swim velocity in Sect. 2.2, and by a description of the simulation technique in Sect. 2.3. Our results for the MSD, the comparison to the experimental data, and a discussion of the non-equilibrium long-time active diffusion coefficients are presented in Sects. 3.1 through 3.3. In Sect. 3.4, we compare the swim velocities predicted by ITT in combination with ABP-MCT to those obtained from computer simulation, before concluding in Sect. 4.

## Methods

### Mode-coupling theory

The ABP-MCT for the description of the collective dynamics in dense ABP systems has been derived in Ref. [30]. For completeness, we recall the central equations of that theory.

We consider a system of *N* ABP in two spatial dimensions, with positions $\mathbf {r}_k$ and orientation angle $\varphi _k$ ($k=1,\ldots N$). The overdamped active-Brownian equations of motion are then

1a $$\begin{aligned} \mathrm{d}\mathbf {r}_k&=\mu \mathbf {F}_k\,\mathrm{d}t+\sqrt{2D_t}\mathrm{d}\mathbf {W}_k+v_0\mathbf {n}(\varphi _k)\,\mathrm{d}t\,, \end{aligned}$$

1b $$\begin{aligned} \mathrm{d}\varphi _k&=\sqrt{2D_r}\mathrm{d}W^\varphi _k\,. \end{aligned}$$

The interaction forces $\mathbf {F}_k$ are approximated to encode hard-sphere interactions, i.e., no two particles are allowed to overlap, and there is no direct interaction among them else. Importantly, they are assumed to be spherically symmetric. The mobility $\mu =\beta D_t$ is chosen to obey detailed balance for the equilibrium passive dynamics (where $v_0=0$, and $\beta =1/kT$ is the inverse temperature). Here, $D_t$ and $D_r$ are the translational and rotational diffusion coefficients, and $d\mathbf {W}_k$ and $dW^\varphi _k$ are component-wise independent Wiener processes that drive the diffusive motion. We fix units of length and time by the typical particle diameter $\sigma$ and $\sigma ^2/D_t$.

The active driving acts along the particle’s orientation vector $$\mathbf {n}(\varphi _k)=(\cos \varphi _k,\sin \varphi _k)^T$$ and is proportional to a self-propulsion velocity $$v_0$$ that is, in this model, fixed per particle. The activity of the ABP is thus controlled by two parameters, the dimensionless self-propulsion velocity $$v_0\sigma /D_t$$ and its persistence time $$D_t/\sigma ^2D_r$$. These parameters are the relevant dimensionless parameters entering ABP-MCT; note that for low-density ABP, the alternative combination of parameters into the Péclet number $$ Pe =v_0^2/2D_rD_t$$ and the persistence length $$\ell _p=v_0/D_r$$ is more natural.

Equation (1) define realizations of a non-Gaussian Markov process whose time-dependent probability distribution function $p(\Gamma ,t)$ in the configuration space $\Gamma =\{\mathbf {r}_k,\varphi _k\}_{k=1,\ldots N}$ is given by the Fokker–Planck (Smoluchowski) equation $\partial _t p(\Gamma ,t)={{\,\mathrm{\Omega }\,}}(\Gamma )p(\Gamma ,t)$. The adjoint Smoluchowski operator (under the ordinary $L^2$-function scalar product) reads

2 $$\begin{aligned} {{\,\mathrm{\Omega }\,}}^\dagger= & {} \sum _{k=1}^ND_t(\mathbf {\nabla }_k+\beta \mathbf {F}_k)\cdot \mathbf {\nabla }_k\nonumber \\&+D_r\partial _{\varphi _k}^2+v_0\mathbf {n}(\varphi _k)\cdot \mathbf {\nabla }_k\,. \end{aligned}$$

In particular, it can be written as ${{\,\mathrm{\Omega }\,}}^\dagger ={{\,\mathrm{\Omega }\,}}^\dagger _\text {eq} +{{\,\mathrm{\delta \Omega }\,}}^\dagger$, to separate the detailed-balance fulfilling equilibrium dynamics and the non-equilibrium perturbation ${{\,\mathrm{\delta \Omega }\,}}^\dagger =v_0\mathbf {n}(\varphi _k)\cdot \mathbf {\nabla }_k$. An alternative splitting that we will encounter in deriving the theory is into the translational and rotational parts, ${{\,\mathrm{\Omega }\,}}^\dagger ={{\,\mathrm{\Omega }\,}}^\dagger _T+{{\,\mathrm{\Omega }\,}}^\dagger _R$ with ${{\,\mathrm{\Omega }\,}}^\dagger _R=D_r\partial _{\varphi _k}^2$.

ABP-MCT starts from the angle-resolved density fluctuations, $\varrho _l(\mathbf {q})=\sum _{k=1}^N\exp [i\mathbf {q}\cdot \mathbf {r}_k] \exp [il\varphi _k]/\sqrt{N}$. The central quantity of the theory is the transient dynamical density correlation function

3 $$\begin{aligned} \Phi _{ll'}(\mathbf {q},t)=\langle \varrho _l^*(\mathbf {q})\exp [{{\,\mathrm{\Omega }\,}}^\dagger t] \varrho _{l'}(\mathbf {q})\rangle \,. \end{aligned}$$

In these correlation functions, the averaging denoted by angular brackets is performed over the equilibrium ensemble, i.e., over the Boltzmann distribution $p_\text {eq}(\Gamma )$ that satisfies ${{\,\mathrm{\Omega }\,}}^\dagger _\text {eq}p_\text {eq}=0$. (Note that in our study of an infinite system in the thermodynamic limit, $p_\text {eq}$ does not depend on the orientations as ${{\,\mathrm{\Omega }\,}}^\dagger _R$ and ${{\,\mathrm{\Omega }\,}}^\dagger _\text {eq}-{{\,\mathrm{\Omega }\,}}^\dagger _R$ commute.) The initial value of the correlation functions is $\Phi _{ll'}(\mathbf {q},t)=S_{ll}(q)\delta _{ll'}$, the matrix of equilibrium static structure factors. Since the orientations of the particles are uncorrelated, we have that $S_{ll}(q)=1$ for all $l\ne 0$. The entry $S_{00}(q)=S(q)$ is the ordinary (hard-sphere) static structure factor known from liquid-state theory of the passive system. These functions and $\Phi _{00}(q,t)$ are isotropic functions of $\mathbf {q}$, setting $q=|\mathbf {q}|$, under the assumption that the system remains statistically homogeneous and isotropic. The correlation functions for $l,l'\ne 0$ obey simple unitary transformation rules under a rotation of $\mathbf {q}$: in particular the quantities

4 $$\begin{aligned} {\tilde{\Phi }}_{ll'}(q,t)=e^{i(l-l')\theta _q}\Phi _{ll'}(\mathbf {q},t) \end{aligned}$$

where $\theta _q$ is the orientation angle of the vector $\mathbf {q}$, do not depend on that orientation.

A Mori–Zwanzig projection operator calculation allows to derive equations of motion for the density correlation functions,

5 $$\begin{aligned}&\partial _t\tilde{\varvec{\Phi }}(q,t)+\tilde{\varvec{\omega }}(q)\cdot \varvec{S}^{-1}(q) \cdot \tilde{\varvec{\Phi }}(q,t)\nonumber \\&\quad +\int _0^tdt'\tilde{\varvec{m}}(q,t-t')\cdot \left( \varvec{1}\partial _{t'} +\tilde{\varvec{\omega }}_R\right) \cdot \tilde{\varvec{\Phi }}(q,t')=\varvec{0}\,.\nonumber \\ \end{aligned}$$

Here, bold symbols refer to matrices in angular-mode indices *l*. The matrix $\omega _{ll'}(\mathbf {q})=-\langle \varrho _l^*(\mathbf {q}){{\,\mathrm{\Omega }\,}}^\dagger \varrho _{l'}(\mathbf {q})\rangle$ is split into its translational and rotational parts, $\varvec{\omega }(\mathbf {q})=\varvec{\omega }_T(\mathbf {q})+\varvec{\omega }_R$ given by

6a $$\begin{aligned} {\tilde{\omega }}_{T,ll'}(\mathbf {q})&=D_tq^2\delta _{ll'}-\frac{iv_0q}{2}S_{ll}(q)\delta _{|l-l'|,1}\,,\nonumber \\ {\tilde{\omega }}_{R,ll'}&=D_rl^2\delta _{ll'}\,. \end{aligned}$$

For the memory kernel, $\tilde{\varvec{m}}(q,t)=\tilde{\varvec{M}}(q,t)\cdot \tilde{\varvec{\omega }}_T^{-1}(q)$, ABP-MCT proposes

7a $$\begin{aligned}&{\tilde{M}}_{ll'}(\mathbf {q},t)\approx \frac{n}{2}\int \frac{d\mathbf {k}}{(2\pi )^2} \sum _{l_3l_4l_{3'}l_{4'}} \tilde{{\mathcal {V}}}^\dagger _{ll_3l_4}(\mathbf {q},\mathbf {k}\mathbf {p})\nonumber \\&\quad \times {\tilde{\Phi }}_{l_3l_{3'}}(k,t){\tilde{\Phi }}_{l_4l_{4'}}(p,t) \tilde{{\mathcal {V}}}_{l'l_{3'}l_{4'}}(\mathbf {q},\mathbf {k}\mathbf {p}) \end{aligned}$$

with $\mathbf {q}=\mathbf {k}+\mathbf {p}$ and vertices $\tilde{\mathcal V}=\tilde{{\mathcal {V}}}^\text {eq}$ and $\tilde{\mathcal V}=\tilde{{\mathcal {V}}}^{\dagger ,\text {eq}} +\tilde{\mathcal V}^{\dagger ,\text {neq}}$, given by

7b $$\begin{aligned} \tilde{{\mathcal {V}}}^{\dagger ,\text {eq}}_{l,mn}(\mathbf {q},\mathbf {k}\mathbf {p})&=e^{il\theta _q}\delta _{l,m+n}\tilde{{\mathcal {Y}}}^{\dagger ,\text {eq}}_{mn}(\mathbf {q},\mathbf {k}\mathbf {p})\,, \end{aligned}$$

7c $$\begin{aligned} \tilde{{\mathcal {V}}}^{\dagger ,\text {neq}}_{l,mn}(\mathbf {q},\mathbf {k},\mathbf {p})&=e^{il\theta _q}\delta _{|l-m-n|,1}S_{ll}(q)\delta _{|l-l'|,1}\nonumber \\&\qquad \times \tilde{{\mathcal {Y}}}^{\dagger ,\text {neq}}_{l-l',mn}(\mathbf {q},\mathbf {k}\mathbf {p})\,. \end{aligned}$$

Here, the coupling coefficients $\tilde{{\mathcal {Y}}}^\dagger$ are determined by the equilibrium static structure factors of the system:

7d $$\begin{aligned}&\tilde{{\mathcal {Y}}}^{\dagger ,\text {eq}}_{mn}(\mathbf {q},\mathbf {k}\mathbf {p}) =D_t e^{-im\theta _k}e^{-in\theta _p}\nonumber \\&\quad \left[ (\mathbf {q}\cdot \mathbf {k})c_m(k) +(\mathbf {q}\cdot \mathbf {p})c_n(p)\right] \end{aligned}$$

with the direct correlation function $c_l(q)=\delta _{l0}c(q)$ given by the usual relation from liquid-state theory, $S(q)=[1-nc(q)]^{-1}$. The non-equilibrium contribution proportional to $v_0$ reads

7e $$\begin{aligned} \tilde{{\mathcal {Y}}}^{\dagger ,\text {neq}}_{l,mn}(\mathbf {q},\mathbf {k}\mathbf {p}) =\frac{iv_0}{2}\delta _{l,m+n}e^{-im\theta _k}e^{-in\theta _p}\nonumber \\ \times \Bigl [ke^{-il\theta _k}S_{l+m,l+m}(k){\tilde{c}}_{m,l+m}(k)\nonumber \\ +pe^{-il\theta _p}S_{l+n,l+n}(p){\tilde{c}}_{n,l+n}(p)\Bigr ] \end{aligned}$$

where we have set ${\tilde{c}}_{ll'}(k)=c_{l}(k)-c_{l'}(k)$.

Equations (5) to (7) form a closed set of nonlinear integral equations that constitute the backbone of ABP-MCT. A similar set of equations holds for the tagged-particle density correlation function [31],

8 $$\begin{aligned} \phi ^s_{ll'}(\mathbf {q},t)=\langle \varrho _l^{s,*}(\mathbf {q}) \exp [{{\,\mathrm{\Omega }\,}}^\dagger t]\varrho _{l'}^s(\mathbf {q})\rangle \,, \end{aligned}$$

where the *N*-particle system is extended to include one additional tracer whose density fluctuations are $\varrho ^s_l(\mathbf {q})=\exp [i\mathbf {q}\cdot \mathbf {r}_s] \exp [il\varphi _s]$. The Mori–Zwanzig equation then reads

9 $$\begin{aligned}&\partial _t\tilde{\varvec{\phi }}^s(q,t)+\tilde{\varvec{\omega }}^s(q)\cdot \tilde{\varvec{\phi }}^s(q,t)\nonumber \\&\quad +\int _0^tdt'\,\tilde{\varvec{m}}^s(q,t-t')\cdot \left( \varvec{1}\partial _{t'} +\tilde{\varvec{\omega }}^s_R\right) \cdot \tilde{\varvec{\phi }}^s(q,t')=\varvec{0}\,.\nonumber \\ \end{aligned}$$

The ABP-MCT memory kernel for the tagged-particle motion is given by $\tilde{\varvec{m}}^s(q,t)=\tilde{\varvec{M}}^s(q,t)\cdot \tilde{\varvec{\omega }}_T^{s,-1}(q)$ with

10a $$\begin{aligned}&{\tilde{M}}^s_{ll'}(q,t)\approx n\int \frac{d\mathbf {k}}{(2\pi )^2}\sum _{l_3l_4} \tilde{{\mathcal {W}}}^s_{ll',l_3l_4}(\mathbf {q},\mathbf {k}) \nonumber \\&\quad \times {\tilde{\Phi }}_{l_30}(k,t){\tilde{\phi }}^s_{l_4l'}(p,t)\,. \end{aligned}$$

In other words, the tagged-particle dynamics can be evaluated once the collective dynamics in terms of the $\varvec{\Phi }(\mathbf {q},t)$ has been determined. The coupling coefficients for the tagged-particle memory kernel are $\tilde{{\mathcal {W}}}^s=\tilde{{\mathcal {W}}}^{s,\text {eq}} +\tilde{{\mathcal {W}}}^{s,\text {neq}}$ with

10b $$\begin{aligned} \tilde{{\mathcal {W}}}^{s,\text {eq}}_{ll',mn}(\mathbf {q},\mathbf {k})&= e^{i(l-l')(\theta _q-\theta _p)}\delta _{ln}\delta _{m0}\tilde{{\mathcal {Y}}}^{s,\text {eq}}(\mathbf {q},\mathbf {k})\,,\end{aligned}$$

10c $$\begin{aligned} \tilde{{\mathcal {W}}}^{s,\text {neq}}_{ll',mn}(\mathbf {q},\mathbf {k})&= e^{i(l-l')(\theta _q-\theta _p)}\delta _{|l-m-n|,1}e^{i(l-n)\theta _p} \nonumber \\&\qquad \times \tilde{{\mathcal {Y}}}^{s,\text {neq}}_{l,mn}(\mathbf {q},\mathbf {k})\,, \end{aligned}$$

and

10d $$\begin{aligned} \tilde{{\mathcal {Y}}}^{s,\text {eq}}(\mathbf {q},\mathbf {k})&={D_t^s}^2(\mathbf {q}\cdot \mathbf {k})^2(c^s(k))^2\,, \end{aligned}$$

10e $$\begin{aligned} \tilde{{\mathcal {Y}}}^{s,\text {neq}}_{l,mn}(\mathbf {q},\mathbf {k})&=D_t^s(\mathbf {q}\cdot \mathbf {k})\frac{ik}{2}e^{-i(l-n)\theta _k}(c^s(k))^2\nonumber \\&\qquad \times \left( v_0^s\delta _{m0}-v_0S(k)\delta _{ln}\right) \,. \end{aligned}$$

For a detailed derivation of this result, we refer to Ref. [37]. In Eq. (10), $c^s(k)$ is the tagged-particle direct correlation function that describes the interactions between a tracer and a host-system particle; in the case of tracer being identical to the host particles that we treat here, $c^s(k)=c(k)$. The peculiar structure in coupling of angular modes stems from the fact that the particles are interacting isotropically. Equations (9) and (10) then are a closed set of integral equations for the tagged-particle correlation functions, given knowledge of the collective dynamics.

From the limit $q\rightarrow 0$ in $\phi ^s_{00}(q,t)\simeq 1-(q^2/4)\delta r^2(t) +{\mathcal {O}}(q^4)$ one obtains the MSD $\delta r^2(t)$ or the tracer particle. Performing this limit in the ABP-MCT equations is an intricate procedure made complicated by the fact that the frequency matrix $\varvec{\omega }_T(\mathbf {q})$ is tri-diagonal and has to be inverted with care in the small-*q* limit. After a tedious calculation, one obtains a set of integral equations to determine $\delta r^2(t)$ and its dipole counterpart $\hat{\phi }^s_{\pm 1,0}(t)=\lim _{q\rightarrow 0}(1/q){\tilde{\phi }}^s_{\pm 1,0}(q,t)$. We get

11 $$\begin{aligned} \partial _t\delta r^2(t)= & {} 4D_t^s-2\sum _\pm (iv_0^s)\hat{\phi }^s_{\pm 1,0}(t)\nonumber \\&-\int _0^tdt'\,{\hat{m}}_{00}^s(t-t')\delta r^2(t')\nonumber \\&+4\sum _\pm \int _0^tdt'\,{\hat{m}}_{0,\pm 1}^s(t-t') (\partial _{t'}+D_r^s)\hat{\phi }^s_{\pm 1,0}(t)\,,\nonumber \\ \end{aligned}$$

and

12 $$\begin{aligned}&(\partial _t+D_r^s)\hat{\phi }^s_{\pm 1,0}(t)=\frac{iv_0^s}{2}\nonumber \\&\quad -2\int _0^t{\hat{m}}^s_{\pm 1,\pm 1}(t-t')(\partial _{t'}+D_r^s) \hat{\phi }^s_{\pm 1,0}(t')\,. \end{aligned}$$

The memory kernels in these equations are given by ${\hat{m}}_{ll'}^s(t)=\lim _{q\rightarrow 0}{\tilde{m}}_{ll'}(q,t)/q^{|l-l'|}$ which are well defined for $|l-l'|\le 1$. An explicit calculation of the memory kernels is given in Refs. [32, 37]. Here, we just point out that to obtain the correct $q\rightarrow 0$ limit of the theory, it is crucial that one recognizes that the $\tilde{\varvec{\omega }}^s_T(q)$ are elements of an infinite-dimensional matrix algebra, since the angular-mode indices $l,l'\in [-\infty ,\infty ]$. The inversion of this matrix which is required to evaluate $\tilde{\varvec{m}}^s(q,t)$ has to be performed on this infinite-dimensional algebra. Only afterward, an angular-momentum cutoff (as required for numerical solutions of the equations) can safely be introduced.

Equations (11) and (12) deserve some discussion. In the low-density limit, all memory kernels ${\hat{m}}_{ll'}^s(t)$ vanish, and one is left with two coupled differential equations that can be solved analytically. One recovers then the familiar result of the MSD of a free ABP,

13 $$\begin{aligned} \delta r^2(t)=4D_tt\left( 1+ Pe \left( 1+\frac{e^{-D_rt}-1}{D_rt}\right) \right) \end{aligned}$$

(dropping *s* superscripts for simplicity). At finite host-system density, for a passive tracer particle Eq. (11) decouples from Eq. (12) as in this case $\hat{\phi }^s_{\pm 1,0}(t)=0$ identically. There appears then a memory kernel ${\hat{m}}_{00}^s(t)$ that is seen to have two contributions: one that reduces to the MCT coupling coefficients in equilibrium, where any self-propulsion of the host-system particles only enters through the activity-enhanced relaxation of the collective density correlation functions. This contribution effectively describes that the passive tracer will experience a renormalized long-time diffusion coefficient that decreases strongly with increasing host-system density and is increased by the host-system activity. There is a second contribution to ${\hat{m}}_{00}^s(t)$ that is directly proportional to the host-particle self-propulsion velocity $v_0$, and this contribution is crucial to obtain also a regime of superdiffusive motion in the MSD that a passive tracer can experience due to interactions with the persistent swimming of the host particles [32].

In the following, we will focus on the case of an active tracer particle in a passive host system, to also connect to recent experiments close to the glass transition [12]. In this case, all three memory kernels appearing in Eqs. (11) and (12) remain relevant.

For our numerical solutions of ABP-MCT, we employ an expression for the static structure factor *S*(*k*) of hard disks that was derived using density-functional theory (DFT) [38]. Since we consider tracer particles that are, in terms of their direct interaction (but not necessarily their self-propulsion) identical to the host particles, we set $c^s(k)=c(k)$. The wave-number integrals are performed on a regular grid with $M=128$ grid points up to a cutoff of $q_\text {max}\sigma =40$. To reduce numerical effort, an angular-mode cutoff $L=1$, such that $l,l'\in [-L,L]$, was introduced. This allows for numerically stable solutions of the ABP-MCT equations up to self-propulsion velocities $v_0\approx 8\sigma /D_t$. Details of the time-domain integration algorithm can be found in Ref. [39].

### Integration through transients

The ABP-MCT with its focus on the transient correlation functions is suited to evaluate non-equilibrium transport coefficients following the ITT approach. ITT was pioneered in the context of shear-driven soft-matter glasses by Fuchs and Cates [40, 41]. A formal integration of the Smoluchowski equation yields for the non-equilibrium stationary average of an observable *A*

14 $$\begin{aligned} \langle A\rangle _\text {neq}=\langle A\rangle _\text {eq} +\int _0^\infty dt'\left\langle \frac{({{\,\mathrm{\delta \Omega }\,}}p_\text {eq})}{p_\text {eq}} e^{{{\,\mathrm{\Omega }\,}}^\dagger t'}A\right\rangle _\text {eq}\,,\nonumber \\ \end{aligned}$$

where $({{\,\mathrm{\delta \Omega }\,}}p_\text {eq})/p_\text {eq}=-v_0\sum _k\beta F_k\cdot \mathbf {n}_k$, writing $\mathbf {n}_k=\mathbf {n}(\varphi _k)$ as a shorthand.

This ITT formula allows to calculate the change in the ensemble average of *A* that is caused by the change in the probability distribution function in response to the non-equilibrium driving term ${{\,\mathrm{\delta \Omega }\,}}$. This makes it ideally suited to address the interaction-renormalization of transport coefficients; however, not the changes caused on a single-particle level as for example the activity-induced extra stresses and pressure terms that occur even if the distribution function remains flat [42, 43].

We specifically evaluate Eq. (14) for the effective swim velocity. The latter is defined by

15 $$\begin{aligned} v(\phi )=v_0+\frac{1}{N}\left\langle \sum _{k=1}^N\mu \mathbf {F}_k\cdot \mathbf {n}_k \right\rangle _\text {neq}\,, \end{aligned}$$

and equivalently $v^s(\phi )$ for a tracer particle. Employing the ITT formula for the second term, one obtains

16 $$\begin{aligned} v(\phi )/v_0=1-\frac{\beta \mu }{N}\int _0^\infty \mathrm{d}t'\left\langle \sum _{jk}\mathbf {F}_j\cdot \mathbf {n}_j e^{{{\,\mathrm{\Omega }\,}}^\dagger t'}\mathbf {F}_k\cdot \mathbf {n}_k\right\rangle _\text {eq}\,.\nonumber \\ \end{aligned}$$

This equation was discussed in detail in the linear-response approximation (where one replaces ${{\,\mathrm{\Omega }\,}}^\dagger$ with the passive-equilibrium time-evolution operator) by Sharma and Brader [44]. Qualitatively, this equation describes how interactions decrease the swim velocity: the positive integral term causes $v(\phi )/v_0\le 1$. However, this form is not yet suited well for approximations at high densities, because such approximations would easily violate the requirement that $v(\phi )/v_0\ge 0$ for symmetry reasons. We thus perform a further exact reformulation akin to the one employed in MCT when rewriting the Mori–Zwanzig equations of motion to an irreducible form: we set

17 $$\begin{aligned} {{\,\mathrm{\Omega }\,}}^\dagger ={{\,\mathrm{\Omega }\,}}^\dagger _\text {irr}+\sum _{kj}\mathbf {F}_k\cdot \mathbf {n}_k \rangle (\beta \mu /N)\langle \mathbf {F}_j\cdot \mathbf {n}_j\,, \end{aligned}$$

and use this splitting of the operator to perform a further Dyson decomposition of $\exp [{{\,\mathrm{\Omega }\,}}^\dagger t]$; see Ref. [37] for details, and also the supplemental material of Ref. [45] for an analogous splitting in the context of particles driven with a fixed force. Setting

18 $$\begin{aligned} C(t)=\left\langle \sum _{jk} \mathbf {F}_j\cdot \mathbf {n}_j\exp [{{\,\mathrm{\Omega }\,}}^\dagger _\text {irr}t'] \mathbf {F}_k\cdot \mathbf {n}_k\right\rangle \,, \end{aligned}$$

a further reduced ITT expression results from Eq. (16):

19 $$\begin{aligned} v(\phi )=\frac{v_0}{1+(\beta \mu /N)\int _0^\infty dt'C(t')}\,. \end{aligned}$$

In this equation, ABP-MCT approximations for *C*(*t*) can be safely applied, and they proceed in analogy to those performed for the memory kernels $\varvec{M}(\mathbf {q},t)$ and $\varvec{M}^s(\mathbf {q},t)$ that govern the density-correlation functions, by projecting the fluctuating forces onto density-pair modes. One gets

20 $$\begin{aligned} C(t)\approx & {} \frac{n}{8\pi }\int dk\,\tilde{{\mathcal {V}}}^{\text {swim}}(k)\nonumber \\&\quad \sum _{l'l''=\pm 1}\Bigl ({\tilde{\Phi }}_{00}(k,t){\tilde{\Phi }}_{l'l''}(k,t)\nonumber \\&\qquad +{\tilde{\Phi }}_{l'0}(k,t){\tilde{\Phi }}_{0l''}(k,t)\Bigr ) \end{aligned}$$

where the vertex $\tilde{{\mathcal {V}}}^\text {swim}(k)$ is given by the equilibrium direct correlation functions [37]. The ABP-MCT with this approximation thus allows to calculate the effective swim velocities of interacting ABP, and (by a suitable extension of Eq. (20)) of an active tracer in an active or passive bath.

### Brownian dynamics simulations

The results of the theory are checked against ED-BD simulation results. This method, described in detail for the passive Brownian system in Ref. [46], and first employed for ABP by Ni et al. [18], is essentially a rejection-free Monte Carlo method to generate valid configurations of hard-sphere (hard-disk) systems. The method consists of segments of the length of a “Brownian time step” $\tau _B$, within each of which an event-driven molecular dynamics simulation is performed to ensure no-overlap conditions among the particles. For this, random velocities are generated from random Gaussian trial displacements such that in the case of a free particle, the correct diffusive motion is generated. To implement self-propulsion, the trial displacements are drawn with an appropriate drift, again such as to ensure that the known analytical results for the free ABP are reproduced.

We employed ED-BD simulations of $N=1000$ slightly size-polydisperse hard disks. Within the parameter ranges and time scales that we study, no signs of crystallization or motility-induced phase separation were observed. After equilibration runs of suitable length, the simulation gives access to the stationary-averaged correlation functions that are the counter-parts of the transient correlation functions obtained from ABP-MCT, and likewise the MSD. Averages over up to 200 independent starting configurations were employed to improve statistics.

For the purpose of the present discussion, we ignore the difference between stationary and transient MSD; the good agreement between theory and simulation (see below) justifies the assumption that for the parameter range that we study here, the two quantities do not differ qualitatively. A similar conclusion was drawn for the case of transient and stationary averages in MCT for sheared passive suspensions [47]. There are systematic differences that can be observed in the off-diagonal elements of the correlation function matrices for times shorter than the rotational persistence time $1/D_r$, as discussed in Ref. [31].

From the simulations, we also extract the average swim velocities. While the ITT expressions mentioned above are justified under the assumption of a smooth pair potential, taking the hard-sphere limit only in the final expression containing the static structure functions, the appearance of the direct interaction forces in Eq. (15) is problematic for the ED-BD scheme. Instead, we obtain the swim velocity directly from the Monte Carlo displacements:

21 $$\begin{aligned} v(\phi )/v_0=\frac{1}{{\mathcal {N}}}\frac{1}{N}\sum _{i=1}^N\sum _{t\in n\tau _B} \frac{\Delta \mathbf {r}_i(t)}{\tau _B}\cdot \mathbf {n}_i(t)\,, \end{aligned}$$

where *n* is an integer number corresponding to an average over a few Brownian time steps and ${\mathcal {N}}$ the corresponding normalization term ${\mathcal {N}}=n v_0$. We found our results to be insensitive to the exact choice of *n*. In Eq. (21), $\Delta \mathbf {r}_i(t)$ is the actual displacement that the ED-BD algorithm assigns to particle *i* in a single Brownian time step. In the case of a free particle, $\Delta \mathbf {r}_i(t)$ contains a passive Brownian contribution that is uncorrelated with the particle orientation, and a term $\propto v_0\tau _B\mathbf {n}_i(t)$, so that in the non-interacting limit $v(\phi )=v_0$ is guaranteed.

## Results

### Mean-squared displacements

A particularly interesting case of the dynamics is that of a single ABP in a dense host system of passive particles. This system has been studied experimentally recently [12].

We have recently discussed the features of the MSD of active and passive tracers in active and passive host systems in a comparison between ABP-MCT and ED-BD simulations [32]. Overall, it was found that the theory describes the simulation results qualitatively correctly. After an empirical rescaling of the density and, in the case of an active host system, the self-propulsion velocity by a global factor of ${\mathcal {O}}(1)$, the agreement is also quantitative for not too large $v_0$. In essence, the theory predicts the slowing down of the dynamics due to approaching glassy arrest at high density, and a rescaling of the density accounts for a numerical error in the predicted value of the glass-transition point ($\phi _c\approx 0.8$ in the simulation compared to $\phi _c\approx 0.7$ in the theory) that is remedied by matching densities in the comparison such that the relaxation times of the passive-particle density-correlation functions agree at a typical wave number corresponding to nearest-neighbor distances ($q\sigma =7$). Close to the glass transition, this amounts in first order to a linear mapping between $\phi ^\text {MCT}$ used in the theory, and $\phi ^\text {BD}$ used in the simulation. Self-propulsion is seen to fluidize the system, so that the transition to the “active glass” gets delayed to higher densities [30]. ABP-MCT somewhat underestimates the effectiveness of self propulsion as compared to the simulations, but otherwise describes it qualitatively. By the same route of matching typical relaxation times, a rescaling $v_0^\text {MCT} =1.5v_0^\text {BD}$ is empirically found [37].

For the free particle, the MSD displays two cross-overs at length scales associated to the persistence of self-propulsion. From the analytical result, Eq. (11), one readily infers these length scales and the associated time scales,

22a $$\begin{aligned} \tau _v&=\frac{4D_t^s}{{v_0^s}^2}\,,&\ell _v&=\frac{2D_t^s}{v_0^s}\,, \end{aligned}$$

22b $$\begin{aligned} \tau _p&=\tau _v(1+ Pe ^s)=\tau _v+\frac{2}{D_r^s}\,,&\ell _p&=\ell _v+\frac{v_0^s}{D_r^s}\,. \end{aligned}$$

For $$t\simeq \tau _v$$, the short-time passive Brownian diffusion (coefficient $$D_t^s$$) crosses over to a superdiffusive regime that is indicative of persistent self-propelled motion. For $$t\simeq \tau _p$$, this persistence is lost, and the free ABP crosses over to enhanced diffusion with coefficient $$D_t^\text {eff}=D_t^s(1+ Pe ^s)$$. Since at low densities these are the only length- and time-scales relevant for the problem, the Péclet number of the tracer, $$ Pe ^s$$ is the only dimensionless number to quantify activity in the long-time limit.

**Fig. 1:**
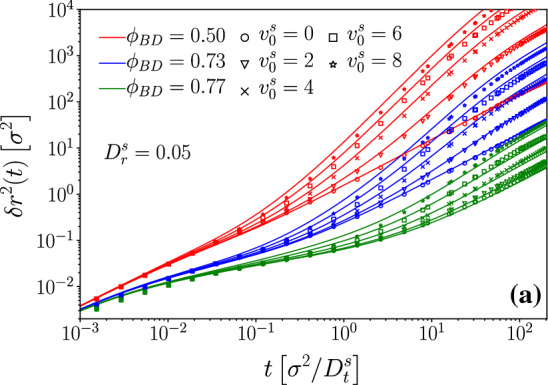
Mean-squared displacement of an active tracer particle embedded in a host system of passive hard disks at packing fraction $\phi$ as labeled, for tracer-self-propulsion speeds $v_0^s$ as labeled, and for fixed reorientational diffusion coefficient $D_r^s=0.05$ and with translational diffusion coefficient $D_t^s=D_t$ equal to that of the host system. Symbols are results from Brownian dynamics simulations, and lines are results from MCT after an adjustment of the packing fraction in order to match the dynamics of the fully passive system (see text)

At high densities, the dynamics of an active particle is characterized by a competition of time and length scales: particle interactions set a length scale of nearest-neighbor cages, $\ell _c$. For $\delta r^2(t)\simeq 4\ell _c^2$, the interactions of the tracer with the host system cause sub-diffusive motion that ultimately leads to dynamical arrest at the glass-transition density. Close to and on the liquid side of the glass transition, an increasing time scale governed by the MCT memory kernels sets the time scale $\tau _\alpha$ on which the sub-diffusive regime ends. ABP-MCT predicts that $\tau _\alpha$ diverges as the glass transition is approached. The cage length scale (of order of $10\%$ of a particle diameter) interferes with the length scales derived from the free-ABP motion. Thus, there emerges a sequence of sub- and superdiffusive regimes in the MSD that depends on the relative magnitudes of the associated time scales, $\tau _c=\ell _c^2/D_t^s$, $\tau _v$, and $\tau _p$, as well as the strongly density-dependent $\tau _\alpha$.

Figure 1 displays as an exemplary case the MSD for an active tracer various self-propulsion velocities $v_0^s\le 8\,D_t/\sigma$ in a passive bath at packing fractions approaching the glass transition. To emphasize the effect of active motion, we have chosen a relatively large persistence time, letting $D_r^s=0.05\,D_t/\sigma ^2$. With these parameters, we obtain for the case $v_0^s=8\,D_t/\sigma$ the relevant time scales as $\tau _c\approx 8\times 10^{-3}\,\sigma ^2/D_t$, $\tau _v=1/16\,\sigma ^2/D_t$, and $\tau _p=(40+1/16)\,\sigma ^2/D_t$, so that $\tau _c<\tau _v\ll \tau _p$.

Correspondingly, the motion in the moderately dense host system ($\phi =0.50$ in Fig. 1) displays essentially a cross-over from short-time diffusion to superdiffusive persistent motion, and at $t\approx \tau _p$ a further cross-over to enhanced diffusive motion. At these densities, the caging influence from the host system is still too weak to be noted dramatically, although a slight sublinear growth in the MSD around $t\approx \tau _c$ can be discerned.

As the host-system density is increased, $$\tau _\alpha $$ increases strongly, and the sub-diffusive regime in the MSD expands over a wider time window. The closer one approaches the glass transition, the more the cross-over to persistent superdiffusive motion is suppressed, so that for the highest density shown in Fig. 1 ($$\phi =0.77$$), superdiffusive motion is no longer evident and the tracer activity essentially serves to provide an enhanced long-time diffusion coefficient as compared to the passive tracer. Note that the enhancement no longer scales with $$ Pe ^s$$: for the parameters used in Fig. 1, the free ABP would show an enhancement of a factor $$ Pe ^s=640$$ for $$v_0^s=8\,D_t/\sigma $$; at $$\phi =0.77$$ the corresponding enhancement is only about a factor 10.

**Fig. 2:**
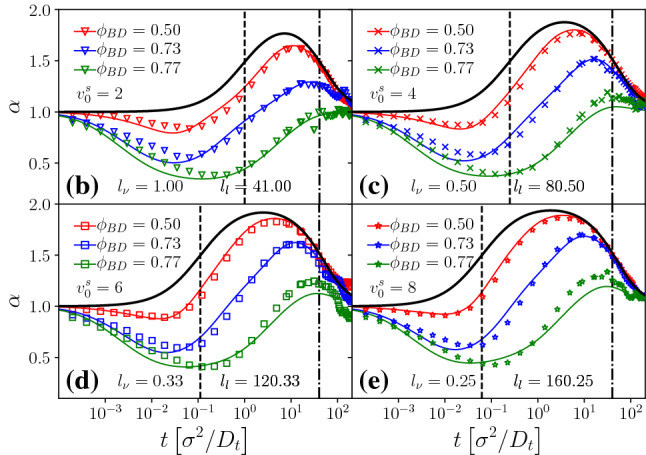
Effective exponents for the mean-squared displacements shown in Fig. 1, obtained from $\alpha (t)=d\log \delta r^2(t)/d\log t$. Symbols are simulation data, lines results from MCT. Solid black lines are the analytical result for the free ABP. Vertical dashed and dash-dotted lines correspond to the free-ABP time scales $\tau _v$ and $\tau _p$, respectively

The sub- and superdiffusive regimes in the MSD and their evolution with time scales are more clearly seen in the temporal evolution of the effective power-law exponent of the MSD, obtained from $\alpha (t)=d\log \delta r^2(t)/d\log t$; these are shown in Fig. 2. One clearly sees a decrease of $\alpha (t)$ to values below unity around $t\approx \tau _c$, reflecting in-cage sub-diffusive motion. The values of $\alpha (t)$ start increasing toward value above unity at $t\approx \tau _v$, and they decrease again toward unity at $t\approx \tau _p$. In order to see truly “ballistic” motion, i.e., $\alpha (t)\approx 2$, even in the free ABP case one would have to separate $\tau _v$ and $\tau _p$ even further, for example, by further decreasing $D_r^s$.

It should be noted that the appearance of superdiffusion in the (stationary) MSD is a clear sign of non-equilibrium dynamics in a system whose governing equations of motion are Markovian-stochastic. ABP-MCT comes at the price of being numerically demanding, but in turn it enables to describe such dynamics correctly; more common and simpler approaches to the glassy dynamics of ABP often proceed by integrating out the orientational degrees of freedom into some effective Smoluchowski operator. It is not evident that with such approximation made from the outset, superdiffusive MSD can be correctly described. Even in the high-density regime where superdiffusion is suppressed, our approach suggests the dynamics to depend on both $v_0$ and $D_r$ as relevant parameters, while simpler approaches typically map these onto a single combined parameter.

### Comparison to experiment

**Fig. 3:**
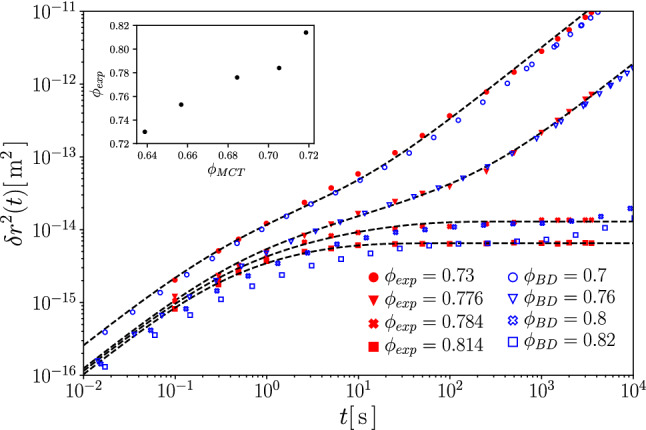
Mean-squared displacement of a passive tracer in a passive host suspension, as obtained from a quasi-two-dimensional experiment [12] (filled symbols), and from our Brownian dynamics simulations (open symbols). Dashed lines indicate our MCT results with the packing fraction adjusted to best describe the simulation data, and after rescaling particle sizes (see text). We find good agreement also between simulation and experiment after a mapping of experimental values for the packing fraction $\phi _\text {exp}$ to that used in simulation, $\phi _\text {BD}$, that is displayed in the inset

In order to compare the recent experimental data of Lozano, Gomez-Solano, and Bechinger [12] with our theory and simulations, we first perform a fit of the fully passive experimental system. This allows to establish a precise enough mapping of packing fractions given in the experiment, and that used in simulation. Differences are expected due to slightly different interaction potentials (experimental particles might not interact as idealized hard spheres), and different size polydispersity (the experimental system uses a binary mixture).

Figure 3 establishes the level of agreement that can be achieved between experiment, simulation, and theory for the passive dynamics. Here, simulation and theory have been shifted by overall factors in time and length; with these adjustments, all three sets of data agree well with each other. There is a deviation notable in the simulation results around $t=1$ whose precise cause we do not know. Also, at times $t > rsim 500$, the Brownian dynamics (BD) data at the highest packing fractions deviate from the idealized arrest curves, most probably due to equilibration issues in the simulation, or due to the appearance of remnant activated relaxation processes (so-called hopping processes).

The rescaling in time scales serves to account for the notable effect of hydrodynamic interactions present in the experimental system. These cause a slowing down already of the short-time passive diffusion that is absent in the simulation and theory. From comparing experimental data at different packing fractions, we estimate that with increasing $\phi$, the experimental $D_t^s(\phi )$ decreases by about a factor 5, in agreement with what is expected from three-dimensional hard-sphere suspensions [48].

The experimental system uses host particles of mean diameter $\sigma \approx {5}\,{\upmu }{\mathrm{m}}$. From the good agreement between MCT and ED-BD simulations for the passive hard-disk system one expects $\ell _c\approx 0.08\sigma \approx {0.4}\,{\upmu }{\mathrm{m}}$ to hold for a system that reasonably well approximates hard-disk behavior, for a tracer of roughly equal size to the host-suspension particles. In the experiment, few Janus particles were added as active tracers with a diameter $\sigma \approx {6.3}\,{\upmu }{\mathrm{m}}$ corresponding to that of the larger particles of the host-suspension mixture. We do not expect the cage-localization length of these particles to be significantly smaller than the above estimate. There remains thus a puzzling effect in the comparison shown in Fig. 3: the experimental data show in-cage localization around $\delta r^2\lesssim 10^{-14}\,{\upmu }{\mathrm{m}^2}$, which corresponds to a localization length $\ell _c^\text {exp}\approx {0.05}\,{\upmu }{\mathrm{m}}$, about a factor of 7 smaller than what is estimated from theory and simulation with the nominal hard-sphere sizes of the particles. The reason for this discrepancy remains unclear; we proceed by adjusting in both simulation and theory an effective diameter $\sigma _\text {eff}=\sigma /7$ of the particles that accounts for this difference. With this adjustment, all passive experimental data are described well. The short-time diffusion coefficient then is read off from Fig. 3 as $D_t\approx {0.008}\,{\upmu }{\mathrm{m}}^2/s$ for the lowest density shown in experiment.

**Fig. 4:**
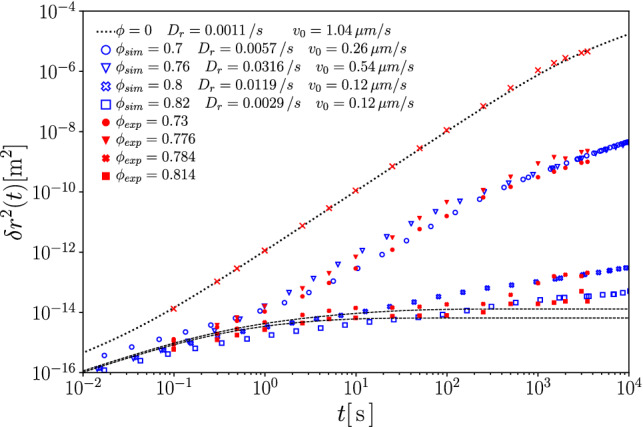
Mean-squared displacements of an active tracer particle in the passive host system at various packing fractions $\phi$ as indicated. Filled symbols are taken from Ref. [12], and they represent the dynamics of a laser-driven Janus particle at fixed laser intensity. Open symbols are Brownian-dynamics simulation results for a hard-disk ABP in a system of passive hard disks, with rotational diffusion coefficient $D_r^s$ as determined from experiment, and self-propulsion velocity $v_0^s$ adjusted to best fit the experimental data. A black dotted line indicates the free-particle solution

For the free Janus particle, Lozano et al. [12] report a self-propulsion velocity of $v_0^s\approx {1.0}\,{\upmu }{\mathrm{m}}/{s}$. Indeed, a fit of the free-ABP MSD, Eq. (13), to the corresponding experimental data yields good agreement with $v_0^s\sigma _\text {eff}/D_t^s=109$; this fit is shown in Fig. 4 as a black dotted line.

Our numerical algorithms to solve the ABP-MCT equations of motion are unfortunately unstable at such large self-propulsion velocities. For this reason, we restrict the further comparison to the experimental data to that with ED-BD simulation results.

A remarkable effect reported in experiment [12] is that the reorientational dynamics of the tracer particle is strongly enhanced as the host-system density approaches the glass transition and then again is suppressed in the glass. This density dependence of $D_r^s$ was attributed to hydrodynamic coupling of the active tracer to the host system via the solvent, and therefore, it is by definition absent in the ABP model system. We model this effect “by hand,” adjusting in the simulations $D_r^s$ to the values reported in experiment.

After this adjustment, the long-time diffusive regime in the experimental MSD can be described from our simulations only after also adjusting the self-propulsion velocity $v_0^s$ as a function of density. The quality of agreement between experiment and simulation is then very good, as displayed in Fig. 4. However, the values required for $v_0^s$ in the simulation show a curious non-monotonic behavior with increasing packing fraction.

The adjusted self-propulsion speed of the tracer drops by a factor of 10 at the highest densities studied, i.e., from about ${1}\,{\upmu }{\mathrm{m}}/{s}$ to ${0.1}\,{\upmu }{\mathrm{m}}/{s}$. This can be rationalized by the expectation that due to deflections from the host-system particles, the self-propulsion mechanism of the tracer in the experiment becomes less effective at fixed energy input with increasing density. This can refer possibly to the laser-energy deposition onto the cap of the Janus particle being less efficient, but also to the fact that the Janus particle requires a region of reversible solvent-phase-separation induced by heating the cap and close to it; the presence of other particles could well perturb this fluid pattern to make self-propulsion less strong. Interestingly, the adjusted $v_0^s$ in the simulation display a maximum at $\phi =0.76$. It is not evident where such a non-monotonic effectiveness of the experimental driving would come from.

This peculiarity aside, both the experiment and the simulation data indicate an interesting effect in the glass: although the host system is, over the time scales that we can access, effectively arrested, the MSD of the active tracer continues to increase beyond the corresponding caging length scale. This could indicate a delocalization transition of a strongly driven active tracer. Such delocalization is known for passive but externally driven particles in a glass; a setup referred to as active microrheology [49]. It is not a priori evident whether the same physical mechanisms applies in both cases: the externally driven tracer is infinitely persistent in its motion if the external force is kept constant, while the ABP has only finite persistence time. Even the limit $D_r^s\rightarrow 0$ might not commute with the limit $t\rightarrow \infty$ taken to decide whether the tracer becomes ultimately delocalized.

### Stokes–Einstein relation

A prominent relation to link the tracer motion to dynamical features of the host system that has been discussed in the context of the glass transition is the generalized Stokes–Einstein (SE) relation. Named after the famous result for the diffusion coefficient *D* of a large colloidal particle moving in a continuum fluid of viscosity $\eta$, $D\eta \sim kT/\sigma$, the SE relation in the context of glassy dynamics refers to the fact that in the fluid regime described by MCT, both the tracer-diffusion coefficient and the host-fluid viscosity are governed by the same cage relaxation processes, and hence show the same control-parameter dependence asymptotically, even if the tracer is of the same type as the particles comprising the host system (and not infinitely larger as in the original SE relation).

**Fig. 5:**
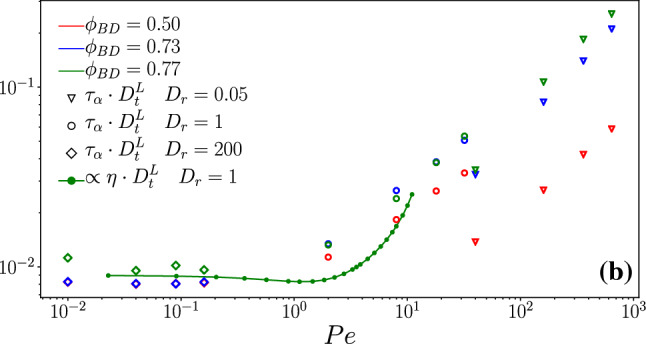
Check of a generalized Stokes–Einstein relation between the active tracer long-time self-diffusion coefficient $$D^L_t$$ and the structural relaxation time $$\tau _\alpha $$ of the active host system. The product $$D^L_t\tau _\alpha $$ is shown for various simulations with different $$D_r$$ and at different packing fractions (symbols and colors as labeled), and for MCT with $$D_r=1=D_r^s$$ (line; for a state close to the glass transition) as a function of Péclet number $$ Pe =v_0^2/2D_rD_t$$

MCT explains the appearance of a SE relation in this sense; but it is also considered one of the theory’s greatest failures to not predict the violation of the SE relation that is observed in computer simulation for densities very close to or above the MCT transition point. Such violations are seen as indicative of relaxation processes “beyond” MCT.

The SE relation is also the basis of the technique of passive microrheology that aims to assess the rheological properties of the host suspension by monitoring the displacement dynamics of an embedded tracer particle. Microrheology has advantages over conventional rheology when providing a sufficient amount of host-suspension fluid is not feasible, and this makes it an interesting technique in particular in the context of biofluids.

It is therefore interesting to check the applicability of the generalized SE relation in the active system. In the spirit of the theory, we extract from our computer simulations the quantity $D^L_t\tau _\alpha$, were $D^L_t$ is the long-time self-diffusion coefficient of an active tracer in an active host suspension whose structural relaxation time (as measured through the density correlation functions at finite *q* close to the main peak of the static structure factor) is $\tau _\alpha$. Figure 5 shows the result for various densities, in simulations with different persistence times $1/D_r^s=1/D_r$, as a function of Péclet number. We observe that in the strongly active system, the product $D\tau _\alpha$ increases by more than an order of magnitude, and there is also a non-trivial $D_r$- and density dependence. Results from ABP-MCT are also shown (lines in Fig. 5); for these we have evaluated the ITT expression for the linear-response viscosity of the active host system [37] to calculate $D^L_t\eta$. In the comparison with simulation, we have made use of the fact that the Green–Kubo integral that determines the viscosity $\eta$ is, close to the glass transition, dominated by a constant (the plateau value of the dynamical correlation function in the cage regime) times the structural relaxation time $\tau _\alpha$, so that a constant rescaling can be performed to match the “proper” $D^L_t\eta$ to the more common $D^L_t\tau _\alpha$ obtained in the simulation. In the comparison we have also included a rescaling of the numerical value of $v_0$ used in the theory, $v_0^\text {MCT}=1.5v_0^\text {BD}$; this accounts for a known underestimation of the strength of the self-propulsion forces in the MCT vertex quantifying the collective relaxation of density fluctuations and has been establishes in detail in comparison to simulations of the density correlation functions [31]. The ABP-MCT curve for $D_r=1$ then qualitatively agrees with the simulation data; in particular, the theory captures the increase of $D^L_t\tau _\alpha$ with increasing $Pe$.

This result emphasizes that the appearance of a generalized SE relation in MCT is not trivial. While the fact that both 1/*D* and $\eta$ asymptotically follow the same power law close to the glass transition in the theory ensures that the product $D\eta$ approaches a constant; however, the fact that this product results in an order of magnitude that is comparable with simulation and moreover indicates a reasonable “effective hydrodynamic radius” of the tracer particle, is in a sense a numerical coincidence.

Qualitatively, the increase with $Pe$ that is seen in Fig. 5 can be rationalized: as the tracer particle becomes more active, it will find it easier to diffuse and hence it will appear effectively “smaller” as long as the collective speeding up of the host-system relaxation is less strong. Also, increasing the persistence length of the tracer particle leads to more effective diffusion as measured through the generalized SE relation.

### Swim velocities

As a genuinely non-equilibrium transport coefficient, the effective swim velocity $v(\phi )$ of ABP plays a significant role. It describes the density-renormalized propulsion, recognizing the fact that the bare self-propulsion velocity $v_0$ of a free ABP is reduced due to interactions with the host-system particles. We next asses the ITT formula Eq. (19) with the specific ABP-MCT closure, Eq. (20), in comparison with computer simulation.

**Fig. 6:**
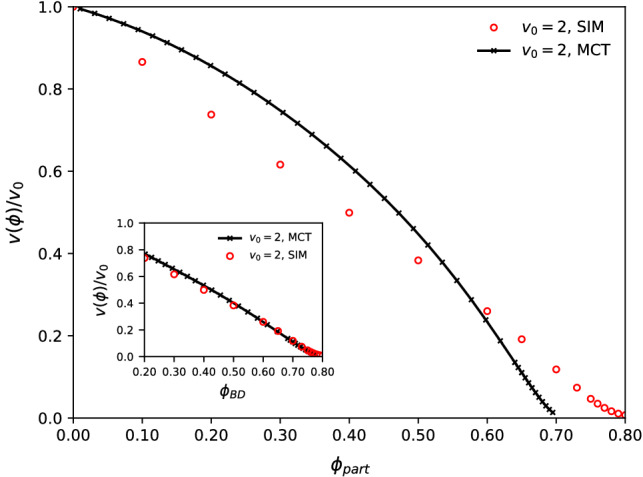
Effective swim velocity $v(\phi )$ of interacting active Brownian particles, in units of the bare self-propulsion velocity $v_0=2$ of a single ABP, at packing fraction $\phi$. Symbols are from BD computer simulations. A line indicates parameter-free results from MCT. The inset shows the same data, but with the density in the MCT calculations adjusted to provide a good description of the relaxation time of the passive system

Figure 6 shows the results from computer simulation and from ABP-MCT for the density-dependent swim velocity in a fully active system at a range of packing fractions spanning from the dilute system to the glass transition. As expected, the swim velocity in the BD simulations is seen to decrease monotonically as interactions become more important with increasing density. At the glass transition, long-range motion of the particles ceases, and this implies that also the swim velocity decays to zero.

As has been noted in similar simulations of soft-core systems before [15, 44] the swim velocity almost follows a linear decrease with increasing density, although closer inspection shows that in particular at high densities there are some deviations from a linear law. The linear law is also what has been assumed in continuum models to study MIPS [33].

The ABP-MCT results at first sight are quite different. The theory correctly predicts a monotonic decay with increasing density, and the fact that the swim velocity approaches zero at the glass transition. But the quantitative agreement with simulations is not optimal. One should, however, note that in this direct comparison, no adjustment of parameters in the theory has been made; in particular, as discussed above in connection with the MSD, one should expect that the theory results need to be compared to simulations at a slightly different packing fraction in order to account for the known numerical error of MCT in prediction the value of the glass-transition point $\phi _c$. In fact, it is instructive to readjust the packing-fraction axis such that the *passive* MCT for each value of $\phi _\text {BD}$, quantitatively matches the relaxation time of the density correlation functions. While close to $\phi _c$ this results in a linear shift of packing fractions, outside the asymptotic regime, quadratic terms in $\phi _\text {MCT}(\phi _\text {BD})$ are needed.

Letting aside the question of how to justify such mapping in detail, it allows us to disentangle two very different effects: one of the quality of the MCT factorization in describing the cage effect, and another one of the quality of the ITT application joint with ABP-MCT in the formula for the swim velocity. Indeed, after adjusting the theory to match the passive relaxation dynamics, also the swim velocities are in rather good quantitative agreement with our simulations, as demonstrated in the inset of Fig. 6.

**Fig. 7:**
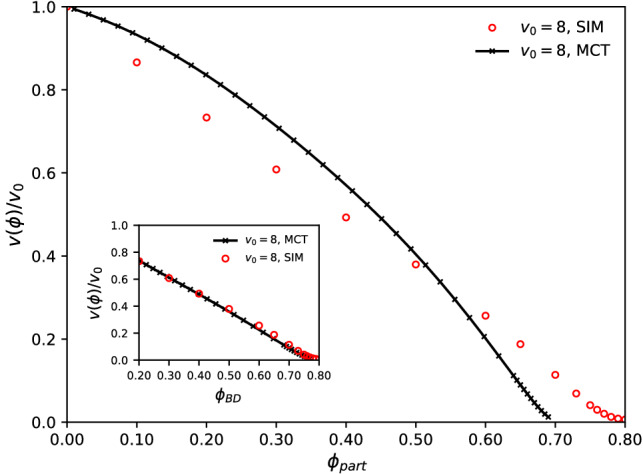
Effective swim velocity *v* of a single ABP tracer in a passive host suspension of packing fraction $\phi$, in units of the tracer’s bare self-propulsion velocity $v_0^s=8$. Symbols are computer simulation results, lines are from MCT. The inset shows the same comparison with a density axis that is adjusted from fitting MCT to the passive host system’s relaxation dynamics

A qualitatively similar finding also holds for the case of a single ABP tracer particle that is embedded in a passive host suspension, shown in Fig. 7. Here, the theory predicts—after the mapping of densities described above—the simulation results quantitatively up to swimming speeds of around $v_0^s=8$; higher values are currently out of reach for the numerical algorithm solving the MCT equations of motion. For the active bath, a velocity of $v_0=8$ results in a qualitatively similar curve for $v(\phi )$, but here, somewhat stronger deviations are seen between theory and simulations at medium densities.

The striking observation is that without parameter adjustment, ABP-MCT predicts the swim velocity to decay initially quadratically with increasing density, not linearly. This is a feature of the way the MCT approximation is constructed: the fluctuating interaction forces are expressed through terms that are quadratic in the fluctuating densities. At low $\phi$, this is not adequate. As a result, already for the fully passive system MCT predicts a wrong density dependence of the relaxation times of the density-correlation functions in the low-density regime; this is of course not usually discussed because MCT is explicitly made for high densities. But it is interesting to note that the reduced ITT formula for the swim velocity, Eq. (19), even with its MCT-like closure seems to introduce no further qualitative errors: if we insert the results from ABP-MCT with packing fractions $\phi ^\text {MCT}$ chosen such that the dynamics of the passive system is well reproduced at any $\phi ^\text {BD}$, the results for the swim velocity are in reasonable agreement. This is observed in the insets of Figs. 6 and 7 where such a density mapping was applied. (It differs from the usually applied linear shift in packing fraction when comparing MCT to simulation by taking into account also leading-order quadratic terms [50].) In essence, the calculation of the swim velocity inherits the deficiency of MCT in the low-density regime, i.e., it is mainly the incorrect density dependence of the low-density relaxation dynamics of density fluctuations that also leads to prima facie incorrect descriptions of the swim velocities.

## Conclusion

We have reviewed the development of the mode-coupling theory for active Brownian particles to describe the mean-squared displacements of an active tracer in a glass-forming host system. The theory was shown to compare favorably to computer-simulation results for the hard-disk system, at moderate Péclet numbers (up to $Pe \approx 640$). It describes the sequence of sub- and superdiffusive motion observed in the MSD of ABP embedded in a dense host system and rationalizes it as arising from a competition of the time scales of nearest-neighbor caging with those of persistent motion.

A direct comparison between ABP-MCT and current experimental data on the self-propelled tracer motion of Janus particles in a glass-forming colloidal suspension was unfortunately not possible. Numerical instabilities in solving the ABP-MCT equations of motion prevent us from addressing the regime of extremely strong self-propulsion. (From the experimental data, one estimates $$ Pe ^s > rsim 10^3$$ at intermediate densities.) It remains to be seen whether improved numerical schemes and/or computational efforts to increase the angular-mode cutoff employed in the numerics will remedy this situation.

However, we have been able to directly compare ED-BD simulations for active-tracer motion in a host suspension of passive hard disks with experiment. It appears that in experiment, both the rotational diffusion and the self-propulsion velocity of the active tracer depend sensitively on the vicinity to the host-suspension glass transition. These effects are not included in the common ABP model of self-propelled particles and complicate the analysis. The strong change in rotational diffusion was noted in experiment directly [12] and attributed to viscoelastic coupling with the host system. If true, this would necessitate an approach where the orientational motion of the ABP tracer couples to the collective density fluctuations of the host system; the development of a fully microscopic theory for this situation needs to be left for future work.

For the regime that is accessible within the theory, non-equilibrium transport coefficients such as the long-time tracer-diffusion coefficient and the effective swim velocity are predicted. One result is the deviation from the commonly assumed Stokes–Einstein relation with increasing $Pe$; it shows that in extracting quantitative information on the host-system viscosity from the long-time diffusivity of a tracer in active fluids, one needs to be careful.

Our comparison with simulation for the swim velocities demonstrates that a decisive factor in predicting these correctly is the correct modeling of the structural relaxation time of the density fluctuation dynamics already in the passive host system. MCT is in quantitative error here, and in particular at low densities it does not yield the correct leading-order variation with packing fraction. This error is often overlooked, because usual comparisons of MCT with simulation or experiment focus on the vicinity of the glass transition, for which the theory was designed. It becomes apparent when calculating for example the density-dependent swim velocity of ABP with the theory: while at high densities, the approach of $v(\phi )$ to zero as $\phi \rightarrow \phi _c$ is correctly captured, the overall shape of the $v(\phi )$-vs-$\phi$ curve is quite different from the almost linear variation that one finds in simulation. We have demonstrated here that this difference does not indicate a failure of the ABP-extension of MCT per se, but rather that ABP-MCT inherits a deficiency from the original MCT that one needs to account for.

The results shown in Figs. 6 and 7 nevertheless confirm a peculiar approach inherent to MCT to such generalized Green–Kubo relations: while the original expression derived using ITT, Eq. (16) describes the reduction of the swim velocity due to interactions by a subtractive mobility term, at high densities an approach that translates the Green–Kubo expression into an additive friction term, Eq. (19). In particular, it allows to explain that the effective swim velocity appears to vanish at the glass transition, as also observed in our ED-BD simulations.

Keeping this in mind, our recent extension of ABP-MCT to include various types of tracer particles is readily generalized to binary mixtures of active and passive particles [37]. This should then provide a promising microscopic theory to address the question how the addition of a few active particles to a passive suspension speeds up the dynamics, or how interacting passive tracers experience enhanced diffusion due to being embedded in an active fluid.
